# AIMP1 downregulation restores chondrogenic characteristics of dedifferentiated/degenerated chondrocytes by enhancing TGF-*β* signal

**DOI:** 10.1038/cddis.2016.17

**Published:** 2016-02-18

**Authors:** J Ahn, H Kumar, B-H Cha, S Park, Y Arai, I Han, S G Park, S-H Lee

**Affiliations:** 1 Department of Biomedical Science, CHA University, Seongnam-si, Gyeonggi-do, Republic of Korea; 2 Department of Neurosurgery, Bundang Medical Center, CHA University, Seongnam-si, Gyeonggi-do, Republic of Korea; 3 Department of Pharmacy, College of Pharmacy, Ajou University, Suwon, Gyeonggi-do, Republic of Korea

## Abstract

Dedifferentiation and degeneration of chondrocytes critically influences the efficiency of cartilage repair. One of the causes is the defect of transforming growth factor (TGF)-*β* signaling that promotes chondrogenic differentiation and degeneration. In the present study, we found that aminoacyl-tRNA synthetase-interacting multifunctional protein 1 (AIMP1) negatively regulates TGF-*β* signaling via interactions with Smad2 and Smad3 in immunoprecipitation assay and luciferase assay. In addition, we observed that the AIMP1 expression level was significantly increased in osteoarthritis (OA) patient-derived degenerated chondrocytes compared with healthy control. So, we hypothesized that downregulation of AIMP1 using small-interfering RNA (siRNA) technology in dedifferentiated (collected at passage #6) and degenerated (obtained from OA-affected areas) chondrocytes could lead to recover TGF-*β* signaling in both chondrocytes. Indeed, AIMP1 downregulation restored TGF-*β* signaling by promoting phosphorylation of Smad2 and Smad3, which shows redifferentiated characteristics in both dedifferentiated and degenerated chondrocytes. Additionally, implantation analyses using *in vivo* mouse model clearly showed that AIMP1 downregulation resulted in the increased chondrogenic potential as well as the enhanced cartilage tissue formation in both dedifferentiated and degenerated chondrocytes. Histological analyses clarified that AIMP1 downregulation increased expression levels of collagen type II (Col II) and aggrecan, but not Col I expression. Taken together, these data indicate that AIMP1 downregulation using siRNA is a novel tool to restore TGF-*β* signaling and thereby increases the chondrogenic potential of dedifferentiated/degenerated chondrocytes, which could be further developed as a therapeutic siRNA to treat OA.

Articular cartilage is an important supportive tissue that covers the ends of bones, reducing the mechanical stress on active joints. Chondrocytes are an unique type of cell that resides in articular cartilage.^1^ Chondrocytes highly synthesize collagen type II (Col II), proteoglycans, and other types of collagens in articular cartilage, and form an integral component of the extracellular matrix (ECM).^2^ Chondrocytes in articular cartilage can be damaged as a result of either traumatic mechanical destruction (automobile accidents and sports injuries) or progressive mechanical degeneration.^3^ Unfortunately, damaged articular cartilage cannot be repaired due to several factors including restricted supply of blood, oxygen, and nutrients^4, 5^ and restricted movement of neighboring chondrocytes to the defect area.^6^ Therefore, several efforts, including cell-based therapies using chondrocytes or mesenchymal stem cells, have been made to repair cartilage defects.^7, 8^ Autologous chondrocyte transplantation (ACT) has gained considerable attention and has renewed interest in cartilage repair. ACT is a promising technique for cartilage repair because autologous chondrocytes are homogeneous and can form hyaline cartilage without immune rejection. In ACT, after the sufficient expansion of chondrocytes by *in vitro* culture, they are implanted into cartilage lesions.^7^ Unfortunately, the number of autologous chondrocytes isolated from a donor is extremely limited; therefore, obtaining a large quantity of chondrocytes that maintain chondrogenic characteristics is an important challenge for ACT. Isolated chondrocytes begin to lose their phenotype upon regular sub-culturing, and this results in serious impairment of their inherent characteristics (from round or polygonal phenotypes to bipolar and fibroblastic phenotypes). Dedifferentiation is a unique feature in chondrocyte biology and is characterized by a comprehensive change in chondrocyte synthetic profile.^9^ Furthermore, a biphasic chondrocyte phenotype shift during dedifferentiation could be observed with a decline in Col II, aggrecan, fibromodulin, and Sox9 expression^10, 11^ and an increase in versican, decorin, and fibronectin expression.^9, 12, 13^

Gene therapy and cartilage tissue engineering have been cooperatively used in cartilage repair.^11, 14^ Transforming growth factor (TGF)-*β* regulates a wide variety of cellular activities and maintains the standard properties of chondrocytes. Of the several TGF-*β* signaling cascades, the TGF-*β*-Smad pathway is most extensively studied in chondrocytes.^15^ Upon TGF-*β* activation, Smad2 and Smad3 are phosphorylated followed by Col II upregulation, which promotes chondrogenic differentiation. However, transduction of TGF-*β*1 signals through Smad2 and Smad3 phosphorylation is perturbed in dedifferentiated chondrocytes due to the decreased sensitivity of TGF-*β* receptors.^16^

Aminoacyl-tRNA synthetase-interacting multifunctional protein 1 (AIMP1, also known as p43) is ubiquitously expressed and has a significant role in many cellular processes including inflammation,^17, 18^ angiogenesis,^19^ would healing,^20^ and glucose homeostasis.^21^ AIMP1 inhibits Smad2 and Smad3 phosphorylation and downregulates TGF-*β* signaling.^22, 23, 24^ Interestingly, AIMP1 is reportedly a component of a negative feedback loop of TGF-*β* signaling. Mouse embryonic fibroblast (MEF) of AIMP1-knockout mice reportedly exhibit increased phosphorylation of Smad2 and Smad3, and knockdown of AIMP1 using short-interfering RNA (siRNA) promotes phosphorylation of Smad2 in human lung adenocarcinoma A549 cells.^22^

The role of AIMP1 in the regulation of chondrocytes has not been studied. In this study, we investigated the role of AIMP1 and attempted to elucidate the mechanism via which AIMP1 elicits effects in dedifferentiated and degenerated chondrocytes. On the basis of the previous reports, we hypothesized that downregulation of AIMP1 in dedifferentiated and degenerated chondrocytes restores chondrogenic characteristics by upregulating Smad2 and Smad3 phosphorylation followed by expression of chondrogenic markers such as Col II. To test our hypothesis, we treated dedifferentiated chondrocytes with AIMP1-targeting siRNA and assessed chondrogenic redifferentiation *in vitro* and cartilage tissue formation *in vivo*. Furthermore, we assessed the role of AIMP1 in degenerated chondrocytes isolated from osteoarthritis (OA) patient.

## Results

### AIMP1 negatively regulates TGF-*β* signaling via its interactions with Smad2 and Smad3

**Figure 1:**
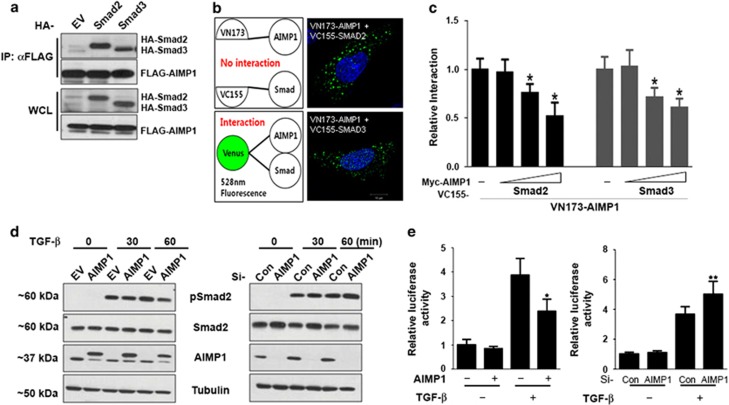
AIMP1 inhibits TGF-*β*1 signaling by associating with SMAD2 and SMAD3. (**a**) HEK293 cells were transfected with FLAG-VN173-AIMP1 and HA-VC155-SMAD2/3. Cell lysates were subjected to immunoprecipitation using an anti-FLAG antibody and blotted with an anti-HA or anti-FLAG antibody. EV, empty vector; WCL, whole-cell lysate. (**b** and **c**) For BiFC analysis, HEK293 cells were transfected with FLAG-VN173-AIMP1 and HA-VC155-SMAD2/3 in the presence or absence of myc-AIMP1. Fluorescence images were captured by confocal microscopy (*, *versus* myc-AIMP1-/HA-VC155-/VN173-AIMP1+). Data represent mean±S.D. of three independent experiments. (**d**) HEK293 cells were transfected with an empty vector (EV) or myc-AIMP1 for 24 h. For knockdown experiment, control or AIMP1 siRNA (20 nM) was transfected for 48 h. Then, TGF-*β*1 (1 ng/ml) was added as indicated. Cell lysates (30 *μ*g) were subjected to SDS-PAGE and blotted with the indicated antibodies. Tubulin was used as an internal loading control. (**e**) SBE4-Luc and the *Renilla* luciferase vector were co-transfected into HEK293 cells in the presence or absence of the AIMP1 for 12 h. To assess knockdown effect of AIMP1, HEK293 cells were transfected with SBE4-Luc and the *Renilla* luciferase vector in the presence or absence or AIMP1 siRNA.Then, cells were treated with TGF-*β*1 (1 ng/ml) for 24 h. Luciferase activity was measured with the Dual-Luciferase Reporter Assay System and normalized against *Renilla* luciferase activity (*, *versus* AIMP1-/TGF-*β*1; **, *versus* si-con/TGF-β1). Data represent mean±S.D. of three independent experiments. *P<0.05 and **P<0.05

In a previous report, AIMP1 was shown to downregulate TGF-*β* signaling via stabilization of smurf2.^22^ In this study, we further assessed the direct interaction of AIMP1 with SMADs. Co-immunoprecipitation assay showed that AIMP1 associates with Smad2 and/or Smad3 (Figure 1a). To further confirm, a bimolecular fluorescence complementation (BiFC) assay was performed as previously described.^25^ This clearly showed that AIMP1 mainly associated with Smad2 and Smad3 in the cytoplasm (Figure 1b). Competition assay using myc-AIMP1 decreased the interaction between FLAG-VN173-AIMP1 and HA-VC155-Smad2/3 in a dose-dependent manner, further confirming the association of AIMP1 with Smad2 and Smad3 (Figure 1c). To unveil the functional relationship, we examined whether the expression level of AIMP1 affected the phosphorylation level of Smad2 via stimulation of TGF-*β*. In the presence of TGF-*β*, overexpression of AIMP1 and knockdown of AIMP1 using siRNA decreased and increased phosphorylation of Smad2, respectively (Figure 1d). In addition, we investigated whether the AIMP1 expression level could affect the transcriptional activity of pSmad2 by stimulation of TGF-*β*. We performed the luciferase activity assay using SBE4-Luc, which contains aSmad-binding element (SBE).^26^ Overexpression and downregulation of AIMP1 decreased and increased TGF-*β*-mediated luciferase activity, respectively (Figure 1e). These data illustrate that AIMP1 negatively regulates TGF-*β* signaling by inhibiting phosphorylation of Smad2 and Smad3.

### AIMP1 downregulation increases phosphorylation of Smad2 and Smad3 in dedifferentiated chondrocytes

**Figure 2:**
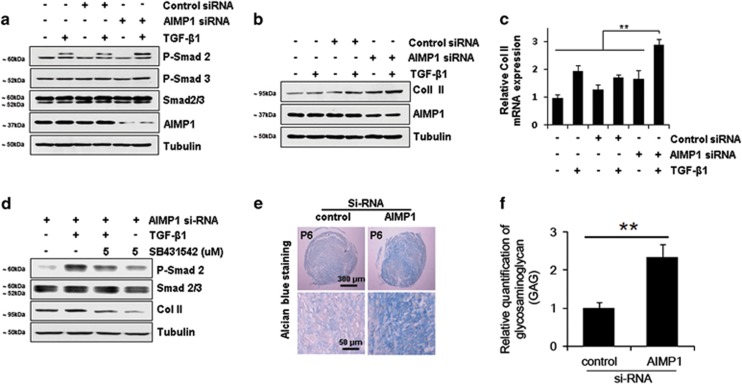
AIMP1 downregulation increases Smad2 and Smad3 phosphorylation and the chondrogenic potential of dedifferentiated chondrocytes. (**a**) Dedifferentiated chondrocytes (P6) were transfected with control or AIMP1 siRNA for 48 h and then TGF-*β*1 (1 ng/ml) was added for 30 min. Phosphorylation of Smad2 and Smad3 was determined by immunoblot analysis. (**b**) The expression of Col II and AIMP1 was examined by immunoblot analysis. Tubulin was used as an internal loading control. (**c**) For qRT-PCR analysis, total RNAs were collected 48 h after TGF-*β*1 treatment and relative Col II mRNA expression was examined. (**d**) SB431542, a specific inhibitor of ALK5, was pretreated as indicated. Phosphorylation of Smad2 and Smad2/3 and expression of Col II were determined by immunoblot analysis. (**e** and **f**) Three-dimensional pellet cultures of cells transfected with control or AIMP1 siRNA in the presence of TGF-*β*1 (1 ng/ml) were stained with Alcian blue, and the Alcian blue-stained area was evaluated in each group. Data represent mean±S.D. of three independent experiments from three individual donors. **P<0.01

It has been known that TGF-*β* signaling is perturbed in dedifferentiated chondrocytes. So, we assessed the feasibility that AIMP1 downregulation using siRNA could restore TGF-*β* signaling in dedifferentiated chondrocytes. Downregulation of AIMP1 indeed increased phosphorylation of Smad2 in dedifferentiated chondrocytes (Figure 2a and Supplementary Figure 1A). In addition, the Col II expression level was increased after treatment with AIMP1 siRNA (Figure 2b and Supplementary Figure 1B). To examine whether the increase in Col II expression was enhanced at the transcription level, we performed quantitative reverse transcription (RT)-PCR analysis. Col II mRNA expression was significantly increased in cells treated with AIMP1 siRNA in the presence of TGF-*β* compared with control siRNA (Figure 2c). In addition, we examined whether enhanced Col II expression was due to increased phosphorylation of Smad2 and Smad3. SB431542, a potent and specific inhibitor of the TGF-*β* superfamily type I activin receptor-like kinase (ALK) receptor, decreased Col II expression in dedifferentiated chondrocytes (Figure 2d), which suggest that the increase in Col II expression following AIMP1 downregulation is due to enhanced TGF-*β* signaling. To test the feasibility of the chondrogenic differentiation of dedifferentiated chondrocytes by AIMP1 downregulation, the intracellular glycosaminoglycan (GAG) content was examined by Alcian blue staining. AIMP1 knockdown distinctly increased GAG matrix formation in dedifferentiated chondrocytes, but not in passage #3 (P3, population doubling (PD) of 6) chondrocytes (Figure 2e and Supplementary Figure 1C and D). The quantitation using ImageJ exhibited that GAG matrix formation was significantly increased in cells treated with AIMP1 siRNA than in cells treated with control siRNA, an increase of approximately 1.6-fold (Figure 2f). These results suggest that AIMP1 silencing is critical to restore TGF*-β*-Smad signaling and favors chondrogenic marker expression in dedifferentiated chondrocytes.

### AIMP1 downregulation increases the chondrogenic potential of dedifferentiated chondrocytes via enhanced nuclear localization of phospho-Smads

Since AIMP1 suppresses Smad2 phosphorylation by TGF-*β* in the cytoplasm as shown in Figure 1, we examined whether subcellular localization of AIMP1 between normal and dedifferentiated chondrocytes shows a different pattern. The expression level of AIMP1 was not changed during the process of dedifferentiation of normal chondrocytes (from P2 to P10) (Supplementary Figure 2A and B). Immunofluorescence staining showed that AIMP1 is mainly localized in the nucleus of normal chondrocytes (P2) (Supplementary Figure 2C). Interestingly, localization of AIMP1 was changed from nucleus to cytoplasm with increasing dedifferentiation (Supplementary Figure 2C and D).

**Figure 3:**
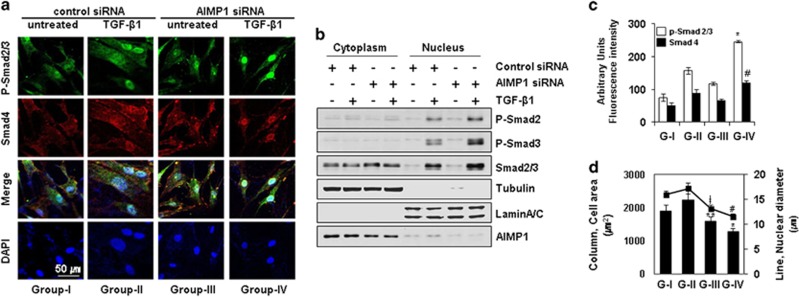
AIMP1 downregulation increased phosphorylation of Smad2/3 and its nuclear translocation in dedifferentiated chondrocytes. (**a**) Control or AIMP1 siRNA was transfected for 48 h and treated with or without TGF-*β* (1 ng/ml) for 30 min. Localization of phosphorylated Smad2/3 and Smad4 was determined by immunofluorescence staining. DAPI was used for nuclear counterstaining. (**b**) The difference in the levels of phosphorylated Smad2/3 between cytoplasmic and nuclear fractions was determined by western blotting. Tubulin was used as an internal cytosolic loading control. LaminA/C was used as an internal nuclear loading control. (**c** and **d**) The fluorescence intensity of phosphorylated Smads was evaluated in nuclei of dedifferentiated chondrocytes (*, *versus* G-II/pSmad2/3; ^#^, *versus* G-II/Smad4), and the cell area and the diameter (* and #, *versus* G-II; ** and ‡, *versus* G-I) were also evaluated. Data represent mean±S.D. of three independent experiments from three individual donors. *P<0.01, **P<0.05, ^‡^*P*<0.05, and ^#^*P*<0.01

To further demonstrate the effect of AIMP1 downregulation on phosphorylation of Smad2 and Smad3, we performed immunoblot and immunostaining analyses of nuclear and cytoplasmic extracts. In dedifferentiated chondrocytes (P6), combined treatment with TGF-*β* and AIMP1 siRNA increased nuclear translocation of Smad4, phosphorylated Smad2 and Smad3 compared with control siRNA/TGF-*β* (Figures 3a and c). Additionally, we determined the area and nuclear diameter of dedifferentiated chondrocytes. The cell area and the nuclear diameter were significantly decreased in the AIMP1 siRNA-treated group in the presence of TGF-*β* (Figure 3d and Supplementary Figure 3A and C). Furthermore, we assessed whether the AIMP1 overexpression could inhibit TGF-*β* signaling in normal chondrocytes. AIMP1 overexpression using adenovirus resulted in the reduced phosphorylation of Smad2/3 by TGF-*β*, and decreased nuclear translocation of Smads (Supplementary Figure 4A and C). Taken together, these results imply that inhibition of AIMP1 expression using siRNA could promote chondrogenic potential of dedifferentiated chondrocytes by restoring TGF-*β* signal.

### AIMP1 downregulation improves cartilage tissue formation of dedifferentiated chondrocytes *in vivo* mouse subcutaneous model

**Figure 4:**
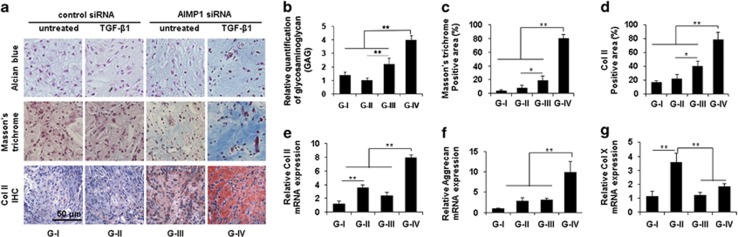
AIMP1 downregulation increased *in vivo* chondrogenesis of dedifferentiated chondrocytes. (**a**) Dedifferentiated chondrocytes were treated as follows: group 1 (G-I), control siRNA; group 2 (G-II), control siRNA/TGF-*β*1 (1 ng/ml); group 3 (G-III), AIMP1 siRNA; group 4 (G-IV), AIMP1 siRNA/TGF-*β*1 (1 ng/ml). Dedifferentiated chondrocytes were transfected with control or AIMP1 siRNA for 48 h and treated with or without TGF-*β*1 for 2 h. Dedifferentiated chondrocytes (1 × 10^6^) along with 200 *μ*l fibrin gel were implanted into the dorsal subcutaneous spaces of mice. At 5 weeks post implantation, tissues were harvested and histological analyses were performed as follows: Alcian blue staining, Masson's trichrome staining, and immunohistochemistry for Col II (**a**). Alcian blue-positive area (**b**), Masson's trichrome-positive area (**c**), and Col II-positive area (**d**) were evaluated. RT-PCR was performed to analyze expression of the chondrogenic marker genes Col II (**e**), Aggrecan (**f**), and ColX (**g**). Bars represent mean±S.D, *n*=4. *P<0.05 and **P<0.01

To validate whether AIMP1 downregulation could promote the redifferentiation potential of dedifferentiated chondrocytes and cartilage tissue formation, we subcutaneously injected P6 chondrocytes (1 × 10^6^) per site of each nude mouse after transfection of control or AIMP1 siRNA in the presence or absence of TGF-*β*, and implants were allowed to develop for 5 weeks *in vivo*. Then, we evaluated the expression of chondrogenic markers and GAG matrix formation. Alcian blue staining, Masson's trichrome staining, and immunohistochemistry for Col II exhibited the enhanced cartilage characteristics of implants when AIMP1 expression was downregulated with siRNA (Figure 4a). Especially, AIMP1 downregulation by siRNA and treatment of TGF-*β* had a synergistic effect because AIMP1 siRNA/TGF-*β*+ (G-IV) showed the highest expression level of GAG matrix formation (Figure 4b) and collagen fiber formation (Figure 4c) compared with siRNA/TGF-*β*− group (G-I), siRNA/TGF-*β*+ group (G-II), and AIMP1 siRNA/TGF-*β*− group (G-III). Interestingly, AIMP1 downregulation using siRNA itself (G-III) showed the increased GAG matrix formation and collagen fiber formation compared with control siRNA/TGF-*β*+ group (G-II) (Figures 4b and c). Immunohistochemical staining of Col II shows agreement with the findings of enhanced GAG matrix and collagen fiber formation (Figure 4d). In addition, RT-PCR analysis showed that AIMP1 siRNA/TGF-*β*+ group significantly increased expression of Col II and Aggrecan, suggesting that the expression was regulated at the transcription level (Figures 4e and f). Expression of ColX, a marker of chondrocyte hypertrophy, was significantly higher in the TGF-*β*-treated group than in the other groups, suggesting that TGF-*β* alone has negative effects on chondrocyte dedifferentiation (P6). Si-RNA-mediated downregulation of AIMP1 alone (G-III) or in combination with TGF-*β* treatment (G-IV) significantly reduced chondrocyte hypertrophy, as assessed by ColX mRNA expression, in comparison with TGF-*β* treatment alone (G-II) (Figure 4g).

### AIMP1 knockdown enhanced expression of chondrogenic markers in OA patient-derived degenerated chondrocytes

**Figure 5:**
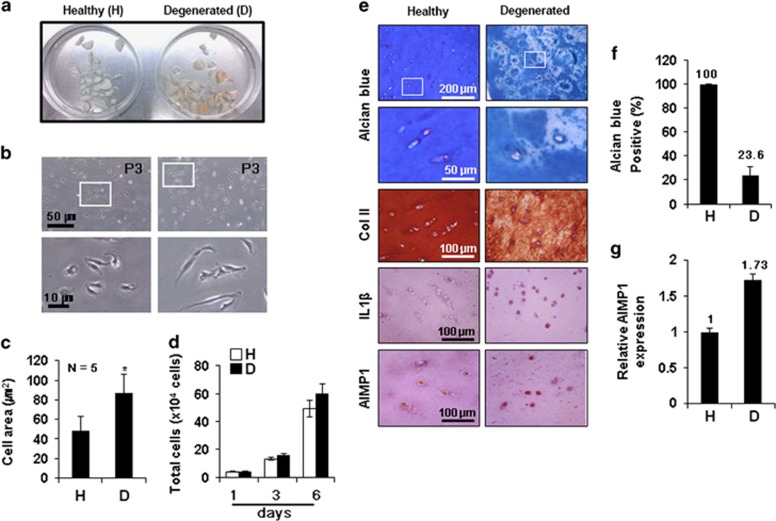
Characterization of healthy and degenerated chondrocytes isolated from OA patients. Articular knee cartilage was obtained by surgery from OA patients with informed consent. (**a**) Morphology of cartilage tissue obtained from OA-affected areas (degenerated) and healthy white cartilage tissue obtained from unaffected areas. (**b**) Chondrocytes were isolated from knee cartilage and subcultured to P3. The morphology of healthy and degenerated chondrocytes was determined by differential interference contrast (DIC) microscopy. Lower panels show magnified images of the boxed regions in the upper panels. Cell area at passage 3 (**c**) and total number of cells (**d**) were evaluated (*, *versus* healthy). (**e**) Col II, IL-1*β*, and AIMP1 expression in chondrocytes within healthy and degenerated cartilage tissues was determined using IHC. GAG matrix formation was determined in ECM (**f**), and AIMP1 expression (**g**) in healthy and degenerated chondrocytes was evaluated. Data represent mean±S.D of three independent experiments from three individual donors. *P<0.05

Next, we also investigated whether the AIMP1 downregulation could have any effect of regeneration in degenerated cartilage tissues isolated from OA patients. First, we isolated healthy and degenerated chondrocytes from OA patients, and characterized. Cartilage tissues obtained from OA-affected areas had inflammatory regions in comparison with healthy white cartilage tissues obtained from the unaffected area (Figure 5a). Isolated OA chondrocytes were significantly larger (2-fold) than healthy chondrocytes (Figures 5b and c). However, the proliferation of cartilage cells isolated from OA-affected areas did not show any difference compared with that of healthy cartilage cells (Figure 5d). Alcian blue staining and Col II immunostaining showed that the expression of GAG and Col II was remarkably reduced in OA-damaged tissue (Figures 5e and f). In particular, Alcian blue staining revealed that GAG matrix formation was decreased about 5-fold in OA cartilage compared with healthy cartilage (Figure 5f). It has been known that interleukin (IL)-1*β*, an inflammatory cytokine, is highly expressed in OA tissues.^27^ IL-1*β* expression was increased at degenerated chondrocyte of OA cartilage tissue compared with healthy cartilage (Figure 5e), in agreement with the previous report.^27^ Interestingly, immunohistochemical staining using AIMP1 antibody showed that AIMP1 was predominantly localized in the nuclei of healthy cartilage, whereas AIMP1 expression was not only increased but also delocalized in both the nucleus and the cytoplasm of degenerated chondrocyte (Figures 5e and g), which shows agreement with the above results (Supplementary Figure 2C and D).

**Figure 6:**
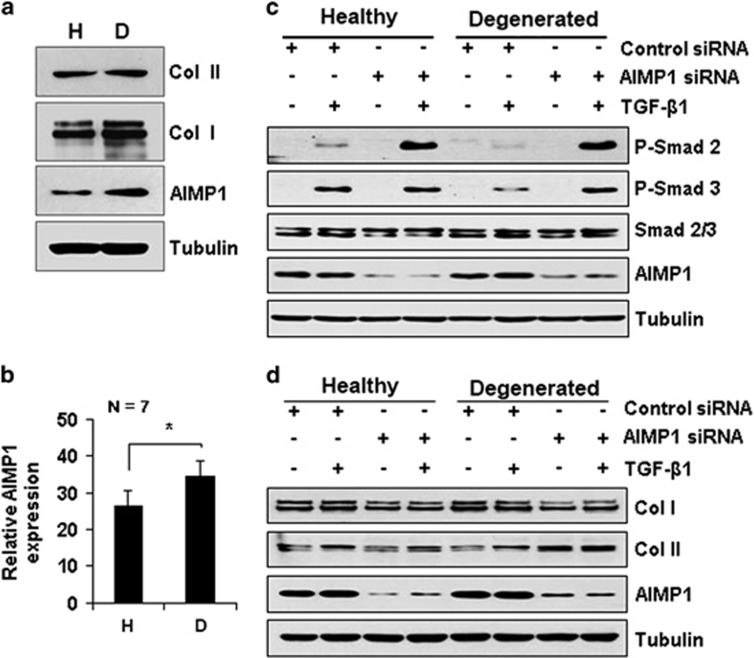
AIMP1 downregulation increases expression of chondrogenic markers in OA patient-derived degenerated chondrocytes. (**a**) Total lysates of healthy (H) and degenerated (D) chondrocytes (P3) were subjected to immunoblot analysis of Col-I, Col II, and AIMP1 expression. Tubulin was used as an internal loading control. (**b**) Difference of AIMP1 expression between healthy and degenerated chondrocytes was evaluated using ImageJ as described in Materials and methods. (**c**) Cells were transfected with control or AIMP1 siRNA for 48 h, and treated with TGF-*β*1 (1 ng/ml) for 30 min. Expression levels of phosphorylated Smad2/3, total Smad2/3, and AIMP1 were determined by western blot analysis. Tubulin was used as an internal loading control. (**d**) In addition, after transfection with control or AIMP1 siRNA for 48 h, TGF-*β*1 (1 ng/ml) was added for 24 h. Then, expression levels of Col I, Col II, and AIMP1 were determined by western blot analysis. Tubulin was used as an internal loading control. Data represent three independent experiments from three individual donors. *P<0.05

To substantiate the role of AIMP1 in pathological conditions, we compared the expression level of chondrogenic markers and AIMP1 between healthy and degenerated chondrocytes by immunoblot analyses. As expected, expression of the osteogenic marker Col I was increased in degenerated chondrocytes compared with healthy chondrocytes, whereas there was no marked difference in Col II expression (Figure 6a). In particular, AIMP1 expression was significantly increased in degenerated chondrocytes (Figures 6a and b). In accordance with dedifferentiated chondrocyte (Figures 1a and 3a and Supplementary Figure 1A), TGF-*β* stimulation with AIMP1 siRNA increased phosphorylation of Smad2 and Smad3 compared with TGF-*β* alone in OA patient-derived degenerated chondrocytes (Figure 6c). To verify the effect of AIMP1 downregulation in degenerated chondrocytes, we examined Col I and Col II expression by immunoblot analysis. In both healthy and degenerated chondrocytes, treatment with AIMP1 siRNA increased the expression level of Col II and decreased the expression level of Col I (Figure 6d).

### AIMP1 downregulation increased cartilage tissue formation of OA patient-derived degenerated chondrocytes *in vivo* mouse subcutaneous model

**Figure 7:**
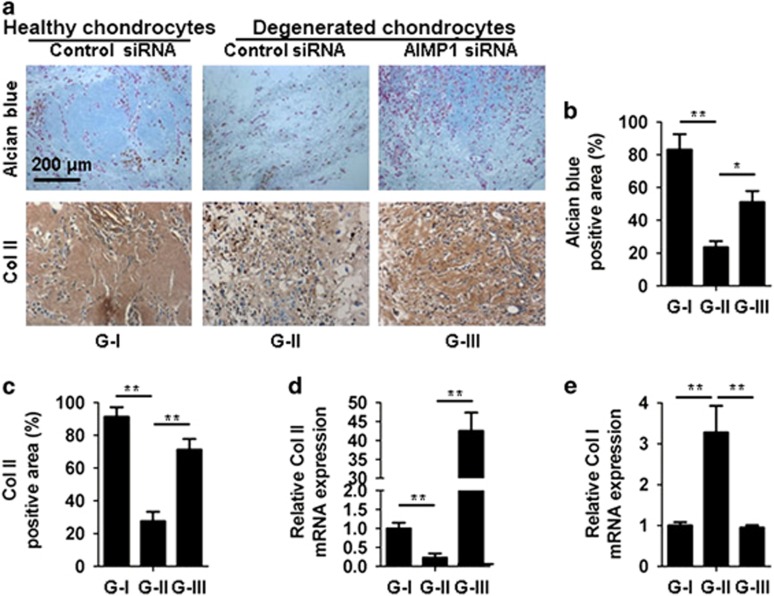
AIMP1 downregulation promotes *in vivo* chondrogenesis of OA patient-derived degenerated chondrocytes. Chondrocytes (P3) isolated from OA patients were transfected with control or AIMP1 siRNA for 48 h and treated with TGF-*β*1 (1 ng/ml) for 24 h. Then, chondrocytes (1 × 10^6^) were implanted along with fibrin gel into the dorsal subcutaneous spaces of mice. The groups were as follows: group 1 (G-I), healthy chondrocytes transfected with control siRNA; group 2 (G-II), degenerated chondrocytes transfected with control siRNA; and group 3 (G-III), degenerated chondrocytes transfected with AIMP1 siRNA. Mice were killed at 5 weeks after implantation, and histological parameters were assessed. (**a**) Alcian blue staining and immunohistochemical staining of Col II as indicated were described in Materials and methods. Alcian blue-positive area (**b**) and Col II-positive area (**c**) were evaluated using ImageJ program. RT-PCR was performed to analyze expression of Col II (**d**) and Col I (**e**). Bars represent mean±S.D., *n*=4. *P<0.05 and **P<0.01

To authenticate the chondrogenic effects of AIMP1 silencing in OA-derived degenerated chondrocytes *in vivo*, expression of chondrogenic markers and GAG matrix formation were evaluated in mouse transplantation model. Consistent with our findings in dedifferentiated chondrocytes (Figure 4), Alcian blue staining and immunohistochemical staining of Col II following implantation of degenerated chondrocytes with AIMP1 siRNA showed an enhanced effect on cartilage tissue formation following chondrogenesis (Figure 7a). For instance, GAG matrix formation (Figure 7b) and Col II-positive area (Figure 7c) were significantly increased in AIMP1 siRNA treatment group (G-III) compared with control siRNA treatment group. Consistent with our findings in dedifferentiated chondrocytes, Col II mRNA expression in degenerated chondrocytes obtained from OA tissue was significantly increased upon AIMP1 downregulation (Figure 7d), whereas the expression of Col I, a chondrocyte dedifferentiation marker, was significantly decreased (Figure 7e).

## Discussion

Current cell-based strategies for ACT require *in vitro* expansion of isolated autologous cells.^28^ The present challenge in ACT is the dedifferentiation of chondrocytes during culturing. Recently, several attempts have been made to prevent the dedifferentiation of chondrocytes, thereby enhancing the efficiency of ACT.^11, 29, 30^ In addition, several growth factors or cytokines can enhance the redifferentiation of dedifferentiated chondrocytes, accompanied by ECM formation.^31, 32, 33^ Nonetheless, the short half-life and high cost of cytokines restrict their use in ACT.

In the present study, we have shown that AIMP1 inhibits TGF-*β* signal through association with Smad2 and Smad3 (Figure 1). Smad2 and Smad3 are phosphorylated by TGF-*β* stimulation, which promotes chondrogenic differentiation via an increase in Col II expression. However, transduction of TGF-*β* signals is perturbed in dedifferentiated chondrocytes.^16^ Therefore, we hypothesized that AIMP1 silencing might influence the redifferentiation of dedifferentiated chondrocytes and increase their chondrogenic potential. Dedifferentiation shifts the production of ECM proteins from chondrogenic (Col II and Agg) to fibroblast specific (Col I).^11, 34, 35^ Additionally, some small proteoglycans such as decorin, biglycan, and the large fibroblast type proteoglycan versican are upregulated during dedifferentiation.^12, 14, 36, 37, 38^ In accordance with the previous reports, we observed analogous effects in dedifferentiated chondrocytes after P6 (Supplementary Figure 5). Previous studies reported that TGF-*β* has a noteworthy role in all phases of chondrogenesis, chondrocyte proliferation, and finally terminal differentiation.^39, 40, 41^ TGF-*β* signals are essential for the repression of articular chondrocyte hypertrophic differentiation.^42^ Furthermore, Smad2 and Smad3 are critical mediators of the inhibitory effect of TGF-*β* on chondrocyte terminal differentiation.^43^ A lack of TGF-*β* or disruption of its signaling pathways results in an cartilage phenotype closely resembling that observed in pathological OA tissue.^44^ Although TGF-*β* has pro-chondrogenic properties,^45^ its presence is not sufficient to completely maintain the articular chondrocyte phenotype and is probably detrimental in cell-based cartilage repair approaches.^44, 46, 47^ Along similar lines, phosphorylation of Smad2 and Smad3 and Col II expression in dedifferentiated chondrocytes were remarkably increased by AIMP1 siRNA with TGF-*β* compared with AIMP1 siRNA or TGF-*β* alone. Furthermore, GAG expression and cartilage formation were significantly increased in dedifferentiated chondrocytes treated with TGF-*β* and AIMP1 siRNA, validating the chondrogenic effects (Figures 2 and 4). Specific receptors, namely, type I and II, are involved in TGF-*β* signal transduction. Among type I receptors, ALK1 and ALK5 have opposite functions in human chondrocytes; Smad-driven effects are inhibited by the former and potentiated by the latter.^48^ In the present study, SB431542, a general ALK5 inhibitor, reduced Col II expression through inhibition of Smad2 and Smad3 phosphorylation, suggesting the importance of Smad2 and Smad3 in chondrogenesis (Figure 2d).

Next, we questioned the relationship between AIMP1 and Smads in chondrocytes. Thus, we investigated the mechanism by which decreased AIMP1 expression increased phosphorylation of Smad2 and Smad3 in dedifferentiated chondrocytes. Previous reports suggest that TGF-*β* signals are transduced into nuclei by Smads.^49, 50^ The localization of AIMP1 changed from the nucleus to the cytoplasm in dedifferentiated chondrocytes (Supplementary Figure 2C and D). This suggests that the role of AIMP1 changes according to its localization in chondrocytes. In particular, AIMP1 is localized in the cytoplasm with prolonged culture, which may affect signaling functions related to the maintenance of chondrocyte characteristics. Treatment with a combination of AIMP1 siRNA and TGF-*β* significantly increased the nuclear, rather than the cytosolic, levels of phosphorylated Smad2 and Smad3 in dedifferentiated chondrocytes (Figure 3). Nuclear localization of Smad2 and Smad3 is important to maintain chondrogenesis.^43^ Therefore, our results suggest that activation of Smads by downregulation of AIMP1 restores chondrogenic characteristics in dedifferentiated chondrocytes (Figures 2, 3, 4).

TGF-*β* signaling is essential for the repression of chondrocyte hypertrophic differentiation and required for maintaining articular cartilage.^42^ Injection of TGF-*β* into the periosteum of rat^51^ or mouse^52^ femur induces chondrocyte differentiation and cartilage formation. These reports suggest the importance of TGF-*β* signaling for chondrogenesis *in vivo*. Despite the pro-chondrogenic properties of TGF-*β*, its presence alone cannot sustain the chondrocyte phenotype. Moreover, TGF-*β* administration during *ex vivo* expansion of human articular chondrocytes redirects the cell phenotype toward hypertrophy.^53^ Previous studies also reported that subsequent to three or four passages, canine,^54^ pig,^55^ and human^56^ chondrocytes reportedly lose their ability to produce cartilage matrix in a nude mouse implantation model. In this study, our results demonstrated that TGF-*β*-mediated phosphorylation of Smad2 and Smad3 is increased in dedifferentiated chondrocytes by downregulation of AIMP1 expression (Figures 2a and 3a). In addition, we confirmed the chondrogenic effects of AIMP1 downregulation in dedifferentiated chondrocytes using an *in vivo* mouse subcutaneous model. Co-treatment of dedifferentiated chondrocytes with AIMP1 siRNA and TGF-*β* not only promoted cartilage formation *in vivo* but also reduced hypertrophy (Figure 4). Thus, our results suggest that AIMP1 downregulation is a novel tool to induce cartilage formation *in vivo* and to resolve the serious problem of hypertrophy.

AIMP1 overexpression suppressed TGF-*β*-mediated phosphorylation of Smad and its nuclear translocation (Figure 1d and Supplementary Figure 4A and C). Interestingly, AIMP1 expression was increased at degenerated chondrocyte of OA cartilage (Figures 5e, g and 6b). It has been known that AIMP1 expression is increased by stimulation of a variety of inflammatory cytokines including TNFα.^20^ TNF*α* as well as IL-1*β* are two major cytokines in the physiopathology of OA.^57^ Therefore, the increased expression of AIMP1 by inflammatory cytokine in OA may induce its translocation from nucleus to cytoplasm to inhibit TGF-*β* signal. However, it needs further study to prove its molecular mechanism. AIMP1 is mainly localized in nucleus of normal chondrocyte, whereas its localization was changed from the nucleus to the cytoplasm with increasing passage number during dedifferentiation process of normal chondrocytes (Supplementary Figure 2C and D). In addition, AIMP1 is mainly localized in the nucleus of OA-derived healthy chondrocytes, whereas its expression was delocalized in both cytoplasm and nuclei of OA-derived degenerated chondrocytes as shown in dedifferentiated chondrocytes (Figure 5e and Supplementary Figure 2C and D). Translocation of AIMP1 from the nucleus to the cytoplasm suggests that AIMP1 inhibits TGF-*β* signaling by interacting with Smad2/3 in cytoplasm as shown in Figure 1. Thus, the increased expression of AIMP1 and its translocation from nucleus to cytoplasm in degenerated chondrocytes provide direct confirmation of the involvement of AIMP1 in the pathogenesis of OA (Figures 5 and 6). Therefore, we assessed whether redifferentiation potential of dedifferentiated chondrocytes by AIMP1 downregulation could be applied to OA-derived degenerated chondrocytes. Col I is typically produced when cells undergo fibroblastic or osteoblastic differentiation.^58^ We found that Col I expression was increased in degenerated chondrocytes compared with healthy chondrocytes (Figure 6a). Treatment with AIMP1 siRNA decreased Col I expression in degenerated chondrocytes both *in vitro* and *in vivo*, suggesting the prospective benefit of AIMP1 downregulation (Figures 6 and 7).

**Figure 8:**
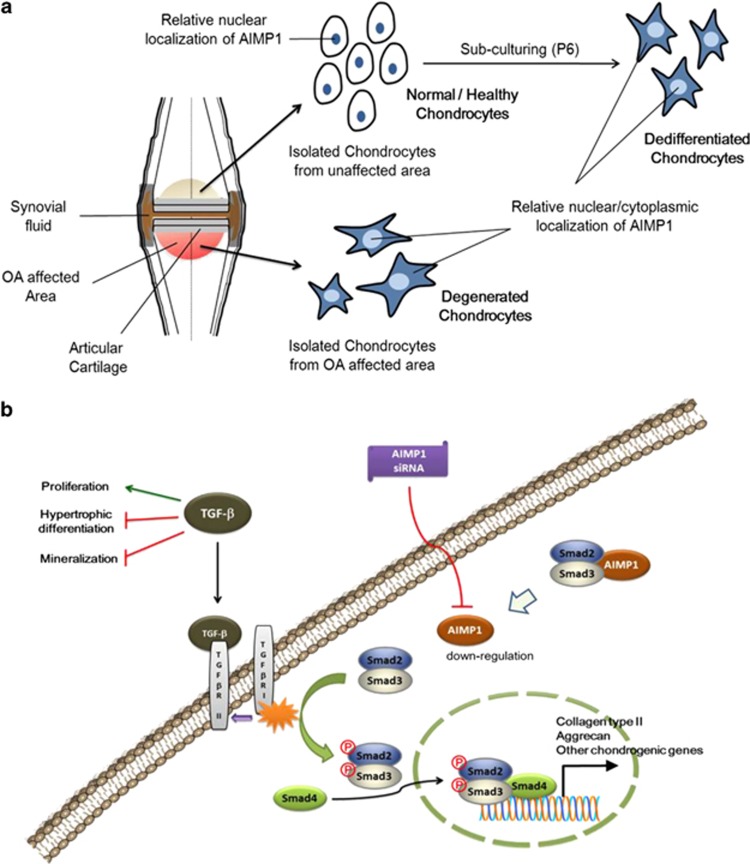
Schematic representation of the role of AIMP1 in dedifferentiated/degenerated chondrocytes. (**a**) AIMP1 localizes in nuclei of healthy chondrocytes. Upon long-term culture (P6), cartilage cells have a fibroblast-like morphology and the localization of AIMP1 in dedifferentiated chondrocytes changes from the nucleus to the cytoplasm. Degenerated chondrocytes isolated from OA-affected areas have a fibroblastic appearance and were similar to dedifferentiated chondrocytes. (**b**) Binding of TGF-*β* to type II receptor (TGF-*β*-RII) leads to recruitment of type I receptor (TGF-*β*-RI) in the highly conserved juxtamembrane region. Activated TGF-*β*-RI then phosphorylates its downstream targets Smad2 and Smad3. Phosphorylated Smads translocate into the nucleus, form complexes with the common mediator Smad4, and upregulate genes that maintain chondrogenic characteristics. AIMP1 negatively modulates TGF-*β* signaling and inhibits phosphorylation of Smads. Downregulation of AIMP1 in dedifferentiated/degenerated chondrocytes using siRNA assists TGF-*β* signaling and facilitates the recovery of chondrogenic characteristics. AIMP1-targeting siRNA treatment increases phosphorylation of Smads and enhances chondrogenic potentials in dedifferentiated/degenerated chondrocytes

A deficiency of Smad3 leads to OA with terminal hypertrophic differentiation of chondrocytes.^42^ Overexpression of both Smad2 and Smad3 blocks the spontaneous maturation of Smad3-deficient chondrocytes.^42, 59^ In the current study, treatment with AIMP1 siRNA and TGF increased phosphorylation of Smad2 and Smad3 in both degenerated and healthy chondrocytes (Figure 6c). Therefore, analogous to dedifferentiated chondrocytes, we confirmed the chondrogenic effects of AIMP1 downregulation in OA-derived degenerated chondrocytes using an *in vivo* mouse subcutaneous model. AIMP1 downregulation increased the chondrogenic potential of OA-derived degenerated chondrocytes *in vivo*, as evidenced by the increased expression of chondrogenic markers such as GAG and Col II (Figure 7). In conclusion, the present study suggests that AIMP1 downregulation using siRNA is a novel tool to restore TGF-*β* signaling, and thus increases redifferentiation and chondrogenic potential of both dedifferentiated and degenerated chondrocytes, which could be further developed as therapeutics to treat OA (Figure 8).

## Conclusions

AIMP1 is mainly localized in the nucleus of normal and healthy chondrocytes. Dedifferentiation and degeneration of chondrocytes induces translocation of AIMP1 from nucleus to cytoplasm, and then AIMP1 inhibits TGF-*β* signaling by associating with Smad2 and Smad3. AIMP1 knockdown using specific siRNA increased the expression of chondrogenic marker and decreased osteogenic marker in both dedifferentiated and OA patient-derived degenerated chondrocytes. Therefore, further development of AIMP1 siRNA may produce an effective drug for the treatment of OA.

## Materials and Methods

### Human chondrocyte isolation and culture

Human articular knee cartilage was obtained by surgery from patients with informed consent, with the approval of the Ethics Committee of CHA Hospital. Primary chondrocytes were prepared from knee cartilage tissue by enzymatic digestion with phosphate-buffered saline (PBS) containing 0.2% (w/v) bovine serum albumin (BSA) and 2 mg/ml collagenase type II (Sigma, St. Louis, MO, USA). Undigested tissue was separated from cells using a 40-mm filter. Cells were centrifuged (1300 r.p.m. for 5 min), washed at least three times, and resuspended in culture media. Freshly isolated chondrocytes were either cultured in cell culture plates for expansion or cryopreserved in liquid nitrogen. Cultures were incubated in low-glucose Dulbecco's Modified Eagle Medium (Gibco BRL, Gaithersburg, MD, USA) supplemented with 10% (v/v) fetal bovine serum (FBS, Gibco BRL) and 100 units/ml penicillin (Gibco BRL) in humidified air with 5% (v/v) CO_2_ at 37 °C. To investigate TGF-*β* signaling, serum starvation for 12 h with 0.5% FBS was performed. At 80% confluency, cells were harvested with 0.5% Trypsin-EDTA (Invitrogen, Grand Island, NY, USA) and subcultured at a seeding density of 1 × 10^4^ cells/cm^2^. For three-dimensional culture, an aliquot of the cell suspension (2 × 10^5^ cells/pellet) was centrifuged at 1300 r.p.m. for 5 min in a 15-ml tube, and the cell pellet was cultured with DMEM supplemented with 10% (v/v) FBS with 100 nM dexamethasone (Sigma) and 10 ng/ml TGF*-β*1 for 3 weeks in humidified air with 5% (v/v) CO_2_ at 37 °C.

### siRNA transfection

Two different siRNA target sequences corresponding to AIMP1 were synthesized (Invitrogen): 5′-AAU CUU CUU CUU AGG AUU CAG CUC C-3′ and 5′-GGA GCU GAA UCC UAA GAA GAA GAU U-3′. For siRNA experiments, dedifferentiated chondrocytes were seeded onto culture plates (2 × 10^4^ cells/cm^2^), and all transfections of siRNA (50 nM) were performed using X-tremeGENE siRNA transfection reagent (Roche Applied Science, Indianapolis, IN, USA) following the manufacturer's instruction. The scrambled control was used at the same final concentration (Dharmacon, Pittsburgh, PA, USA). Two days after knockdown, cells were harvested to investigate downregulation of AIMP1 by western blot analysis.

### RT-PCR and quantitative real-time PCR analysis

Total RNA was extracted from transfected cells using TRIzol reagent (Invitrogen), and 1 *μ*g of total RNA was used for cDNA synthesis with RT-PreMix (Bioneer, Daejeon, Korea). PCR was performed with PCR-PreMix (Bioneer) under standard PCR conditions. The primers used to amplify Col II, ColX, Agg, and glyceraldehyde 3-phosphate dehydrogenase (GAPDH) are provided below. PCR consisted of an initial denaturation step at 94 °C for 1 min, followed by 27 amplification cycles consisting of denaturation at 94 °C for 30 s, annealing at 55 °C for 30 s, and extension at 72 °C for 1 min, with a final extension at 72 °C for 10 min. PCR products were analyzed by ultraviolet irradiation of a 1.2% agarose gel stained with ethidium bromide. For quantitative real-time PCR analysis, gene-specific primers were designed to amplify Col II, Agg, ColX, and the GAPDH as follows: Col II (forward primer, 5′-CAC GTA CAC TGC CCT GAA GGA-3′ reverse primer, 5′-CGA TAA CAG TCT TGC CCC ACT T-3′), Agg (forward primer, 5′-GCC TGC GCT CCA ATG ACT-3′ reverse primer, 5′-ATG GAA CAC GAT GCC TTT CAC-3′), ColX (forward primer, 5′-ACG CTG AAC GAT ACC AAA TG-3′ reverse primer, 5′-TGC TAT ACC TTT ACT CTT TAT GGT GTA-3′), and GAPDH (forward primer, 5′-ACA TCG CTC AGA CAC CAT G-3′ reverse primer, 5′-TGT AGT TGA GGT CAA TGA AGG G-3′). All amplifications were performed in a final reaction mixture (20 *μ*l) containing 1 × SYBR Supermix, 500 nmol/l gene-specific primers, and 1 *μ*l of template using the following conditions: initial denaturation at 94 °C for 10 min, followed by 45 cycles of 94 °C for 10 s, 55 °C for 45 s, and 72 °C for 30 s, with a final extension at 72 °C for 5 min. After amplification, the baseline and threshold levels for each reaction were investigated using the software package of the company (Exicycler 96; Bioneer). For validation, amplified products were separated on 1.2% agarose gels and visualized by ethidium bromide staining.

### Western blot analysis

Cells were washed twice with ice-cold PBS and lysed in RIPA buffer (Sigma) containing a protease inhibitor cocktail to extract proteins. The mixture was incubated at 4 °C for 30 min, and total proteins were extracted by centrifugation at 15 000 r.p.m. for 30 min. Approximately 20 *μ*g of total protein was separated by SDS-polyacrylamide gel electrophoresis (PAGE). Proteins were transferred onto polyvinylidene fluoride membranes using the Trans-Blot semi-dry transfer kit (Bio-Rad, Hercules, CA, USA), followed by blocking for 30 min with 5% non-fat dry milk prepared in Tris-buffered saline (TBS) containing 0.1% Tween-20 (TBS-T), and then incubated with the appropriate primary antibodies diluted in 1% BSA solution at 4 °C for 16 h. The primary antibodies were anti-TGF*β*RII (1 : 500, Abcam, Cambridge, UK), anti-TGF*β*RI (1 : 500, Abcam), anti-Smad1/5/8P (1 : 1000, Cell Signaling, Danvers, MA, USA), anti-Smad2P (1 : 1000, Cell Signaling), anti-Smad2 (1 : 1000, Cell Signaling), anti-Col-I (1 : 1000, Abcam), Agg (1 : 1000, Abcam), Smad3 (1 : 1000, Cell Signaling), Tubulin (1 : 1000, Abcam), Customized AIMP1 (1 : 1000, Hong *et al.*^60^), anti-Col II (1 : 1000, Millipore, Darmstadt, Germany), and Lamin A/C (1 :  1000, Millipore). Membranes were washed four times for 10 min with TBS-T and incubated with horseradish peroxidase-conjugated secondary antibodies (0.1 *μ*g/ml; Santa Cruz Biotechnology, Dallas, TX, USA). Immunoreactive bands were detected using the WEST-one western blotting detection system (iNtRON Biotechnology, Seoul, Korea) according to the manufacturer's instructions.

### Immunofluorescence

Cells grown on circular glass coverslips (VWR LabShop, Batavia, IL, USA) in 24-well plates were fixed with 4% paraformaldehyde for 5 min and then permeabilized with PBS supplemented with 0.1% Triton X-100 for 5 min. After washing with PBS with Tween (PBST) three times for 5 min, fixed cells were incubated for an additional 30 min in PBST containing 1% BSA to prevent non-specific binding of antibodies, and then incubated with specific primary antibodies for 1 h. Cells were washed three times with PBST and then incubated with Alexa 488- and Alexa 594-conjugated goat anti-mouse or anti-rabbit secondary antibodies (Molecular Probes, Eugene, OR, USA). DNA was counterstained with 4', 6'-diamidino-2-phenylindole (DAPI, 0.1 *μ*g/ml prepared in PBST).

### Chondrocyte implantation and *in vivo* cartilage formation

Seven-week-old female athymic mice (BALB/c-nude, Orientbio, Seoul, Korea) were anesthetized by intraperitoneal injection of Zoletil 50 (10 mg/kg; Virbac, Carros, France). A total of 1 × 10^6^ dedifferentiated chondrocytes were implanted per site into the dorsal subcutaneous spaces of mice (*n*=4 per group) with 200 *μ*l fibrin gel (Greenplast Kit, Green Cross, Chungbuk, Korea). Each sample was injected into four groups of four mice. Five weeks after implantation, all mice were killed and all implants were retrieved for histological analysis. All procedures performed on animals followed the guidelines of an approved protocol from the CHA University Institutional Animal Care and Use Committee. Half of each sample was fixed in 4% formalin overnight at room temperature, embedded in paraffin, and sectioned transversely at a thickness of 5 *μ*m. Cartilage tissue formation was evaluated by Alcian blue staining and immunohistochemical staining of Col II. Alcian blue stains GAG in cartilage tissues. The area of cartilage was measured using the NIH ImageJ version 1.34e software (http://rsbweb.nih.gov/ij/) coupled to a light microscope and then quantified as the percentage area (blue staining) of the available pore space and other tissues ((cartilage area/pore and other tissue areas) × 100%). Half of each unfixed specimen was used for RT-PCR analysis of chondrogenic gene expression.

### Histology and immunohistochemistry

Dedifferentiated chondrocytes implanted into the dorsal subcutaneous spaces of mice were fixed with 4% formalin. Blocks of specimens were embedded in paraffin and cut into 5-*μ*m-thick slices. Sections were stained with 0.5% Alcian blue prepared in 0.1 M HCl (pH 1.0) for 1 h and rinsed with tap water for microscopy analyses of GAG in cartilage tissue. For immunohistochemical staining, sections were deparaffinized with xylene for 10 min, followed by sequential hydration in ethanol. After being washed with PBS for 5 min, sections were blocked with 5% BSA for 30 min. Samples were incubated overnight at 4 °C with a primary antibody against Col II (Millipore) and then with an FITC-conjugated goat anti-rabbit antibody (Applied Biological Materials Inc., Richmond, BC, Canada) for 1 h at room temperature. Samples were examined with a Zeiss LSM 510 Meta laser scanning confocal microscope (Carl Zeiss Microimaging Inc., Göttingen, Germany).

### Construction of plasmids and adenoviruses

Expression vectors encoding AIMP1, SMAD2, and SMAD3 were constructed by subcloning the corresponding cDNAs into HA- or FLAG-tagged BiFC plasmids (pFLAG-VN173-CMV or pHA-VC155-CMV) containing fragments derived from the newly engineered fluorescent proteinVenus, which were kindly provided by Chang-Deng Hu (Perdue University, IN, USA). Adenoviruses encoding human AIMP1 (Ad-AIMP1) were generated by insertion of the AIMP1 ORF into pAdTrack-CMV expressing GFP (Addgene, Cambridge, MA, USA).

### Co-immunoprecipitation and western blot analysis

HEK293 cells were transfected with the indicated vectors using Lipofectamine Plus (Invitrogen). Cells were lysed with RIPA buffer (20 mM Tris-HCl, pH 7.6, 150 mM NaCl, 1 mM EDTA, 1 mM EGTA, 1% Nonidet P-40, 0.1% sodium deoxycholate, 0.1% SDS, 10 mM NaF, 0.1 mM Na_3_VO_4_, 12 mM *β*-glycerophosphate, 5 *μ*g/ml aprotinin, and 1 mM PMSF). Cell lysates (300 *μ*g) were incubated with an anti-FLAG antibody (3 *μ*g) for 6 h at 4 °C. Then, protein G agarose (Invitrogen) was added to the reaction mixture for 4 h at 4 °C. Precipitates were washed three times, subjected to 9% SDS-PAGE, and incubated with specific antibodies. Blots were developed using an enhanced chemiluminescence kit (Santa Cruz Biotechnology).

### BiFC analysis

VN173 containing AIMP1 (FLAG-VN173-AIMP1) and VC155 containing SMAD2 or SMAD3 (HA-VC155-SMAD2 or -SMAD3) were co-transfected into HEK293 cells for 24 h, and fluorescence images were captured at 488 nm using a charge-coupled device camera mounted onto a TE2000-U inverted fluorescence microscope (Nikon, Melville, NY, USA) with JP4 filters (Chroma, Rockingham, VT, USA). For the competition assay, FLAG-VN173-AIMP1 was transfected into HEK293 cells along with 0.5 *μ*g of HA-VC155-SMAD2 or -SMAD3 in the presence or absence of Myc-AIMP1(0.1, 0.5, or 1 *μ*g). The BiFC assay was performed at 24 h post transfection. Detectable fluorescence signals were counted using a microscope (Nikon) as described previously.^25^

### Statistics

Data are expressed as mean±standard deviation (S.D.). One-way analysis of variation (ANOVA) was used to compare groups. At least three independent sets of experiments for each condition were performed in triplicate. *P*<0.05 was considered as statistically significant.

## Abbreviations

AIMP1: aminoacyl-tRNA synthetase-interacting multifunctional protein 1
OA: osteoarthritis
siRNA: small-interfering RNA
Col II: collagen type II
ECM: extracellular matrix
ACT: autologous chondrocyte transplantation
TGF: transforming growth factor
BiFC: bimolecular fluorescence complementation
SBE: Smad-binding element
ALK: activin receptor-like kinase
GAG: glycosaminoglycan
