# Supplementary material for: AIMP1 downregulation restores chondrogenic characteristics of dedifferentiated/degenerated chondrocytes by enhancing TGF-β signal
Source: Cell Death Dis. 2016 Feb 18;7(2):e2099–. doi: 10.1038/cddis.2016.17 (PMC5399188; doi:10.1038/cddis.2016.17)
Supplement: Supplementary Information [file cddis201617x1.docx]

**Supplementary Information**

**Supplementary Captions**

*Supplementary Figure1.* AIMP1 knock-down effect in normal and dedifferentiated chondrocytes. (A-B)P3 (normal) or P6 (dedifferentiated) chondrocytes were transfected with control or AIMP1 si-RNA for 48 h. TGF-β1 (1 ng/mL) was treated for 30 min or 24 h to analyze Smad phosphorylation and collagen expression, respectively. Cell extracts were harvested and subjected to immunoblot analysis as indicated. Tubulin was used as an internal loading control. (C-D) *In vitro* pellet culture was performed for 3 weeks, and Alcian blue staining were performed to compare the level of GAG between dedifferentiated and normal chondrocytes treated with control or AIMP1 si-RNA. Alcian blue positive area was evaluated in each group and presented as bar graph. Data represent mean ± SD of three independent experiments.***P*<0.01.

*Supplementary Figure2.* AIMP1 expression and its localization in chondrocytes at different passage numbers. (A-B) Protein extracts from chondrocytes at different passage numbers were subjected to immunoblot, and AIMP1 expression level was presented as bar graph. α-Tubulin was used as an internal loading control. (C-D) AIMP1 localization in chondrocytes at different passage numbers was investigated by immunofluorescence staining with an anti-AIMP1 antibody. DAPI was used for nuclear counterstaining. The nuclear AIMP1 intensity was evaluated and presented as bar graph. Data represent mean ± SD of three independent experiments. **P*< 0.05 and ***P*<0.01.

*Supplementary Figure 3.* AIMP1 down-regulation induces the morphological transformation of dedifferentiated chondrocytes into normal chondrocytes. Primary chondrocytes were isolated from human knee cartilage tissue and sub-cultured every 2–3 days to reach the terminally differentiated stage (P6). Dedifferentiated chondrocytes (P6) were transfected with control or AIMP1 si-RNA for 48 h. (A) Cellular morphology was determined by DIC microscopy (upper panels). Lower panels show magnified images of the boxed regions in the upper panels. (B) The expression of ColII and AIMP1 was assessed by immunoblot analysis. Tubulin was used as aninternal loading control. (C) In addition, the cell area was evaluated in each group and presented as bar graph (n=30). Data represent mean ± SD of three independent experiments. **P*<0.05 and** *P*<0.01.

*SupplementaryFigure 4.* AIMP1 over-expression suppresses phosphorylation of Smad2/3 and its nuclear translocation by TGF-β1 in chondrocytes. Normal chondrocytes (P3) were infected with a mock (Ad-MOCK) or AIMP1 (Ad-AIMP1) adenovirus, and then treated with TGF-β1 (1 ng/mL) for 30 min. (A) Proteins were fractionated into nucleus and cytoplasm to assess the localization of phosphorylated Smads. Phosphorylation of Smad2 and Smad3 was determined by western blot analysis. Tubulin and Lamin A/C were used as cytoplasmic and nuclear markers, respectively. (B) Immunofluorescence staining was performed to examine the localization of phosphorylated Smad2/3 as described in the Methods section. (C) Nuclear fluorescence intensity was evaluated and presented as bar graph (* and #, vs. G-I; ** and ǂ, vs. G-II). Data represent mean ± SD of three independent experiments. **P*<0.01, ***P*<0.05, ^#^*P*<0.01, and^ǂ^ *P*<0.05.

*Supplementary Figure 5.* Characterization of normal and dedifferentiated chondrocytes. Primary chondrocytes were isolated from human knee cartilage tissue and sub-cultured every 2–3 days to reach the terminally differentiated stage (P6). (A) Cellular morphology was compared between P2 and P6 by differential interference contrast (DIC) microscopy. Lower panels show magnified images of the boxed regions in the upper panels. (B) Chondrocytes were cultured as a monolayer in basal medium and harvested for western blot analysis of ColI, ColII, and Aggrecan. (C) TGF-β1 (1 ng/mL) was treated to P2 and P6 chondrocytes for 30 min, and cell lysates were harvested, and immunoblotted with anti-p-Smad2, anti-p-Smad3, anti-totalSmad2/3 antibodies, respectively. Tubulin was used as a loading control. Data represent three independent experiments.
