# Supplementary figures and images for: AIMP1 downregulation restores chondrogenic characteristics of dedifferentiated/degenerated chondrocytes by enhancing TGF-β signal
Source: Cell Death Dis. 2016 Feb 18;7(2):e2099–. doi: 10.1038/cddis.2016.17 (PMC5399188; doi:10.1038/cddis.2016.17)

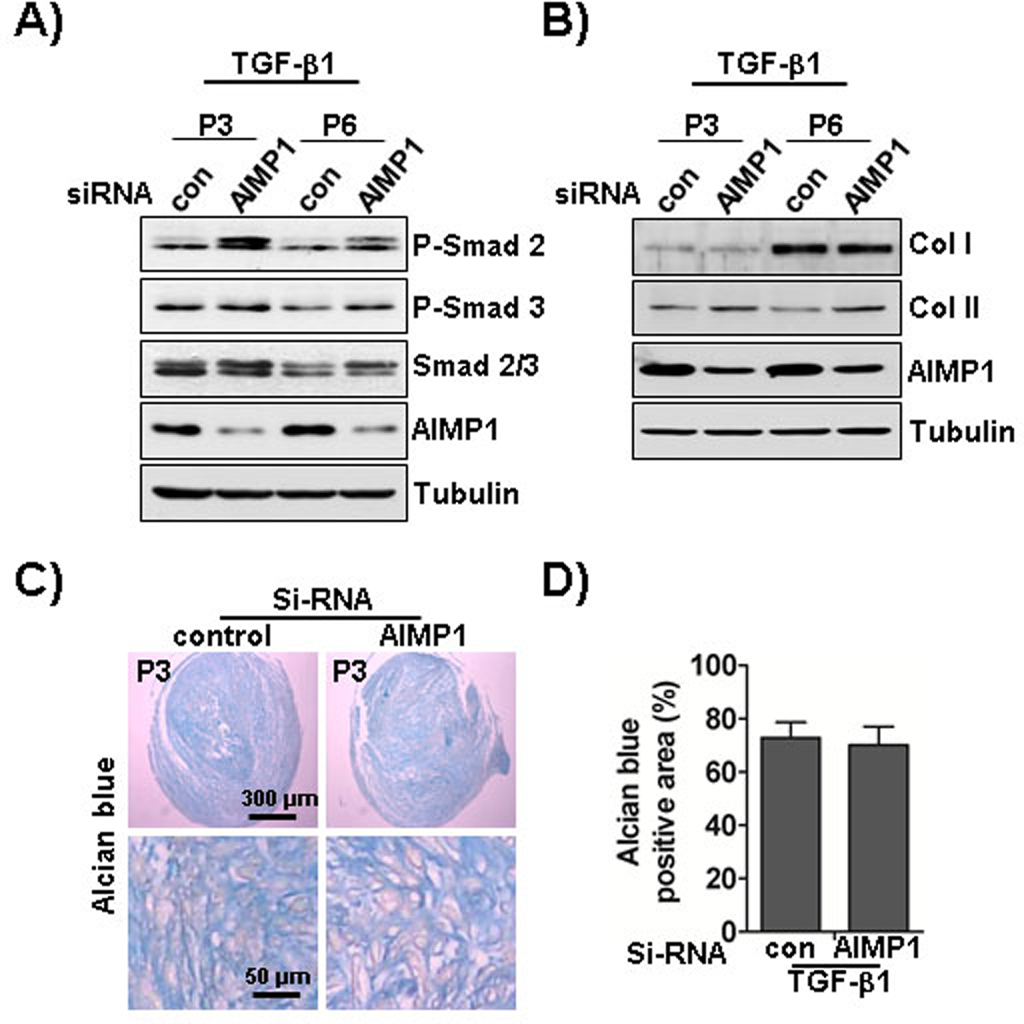

Supplement: Supplementary Figure 1 [file cddis201617x2.tif]

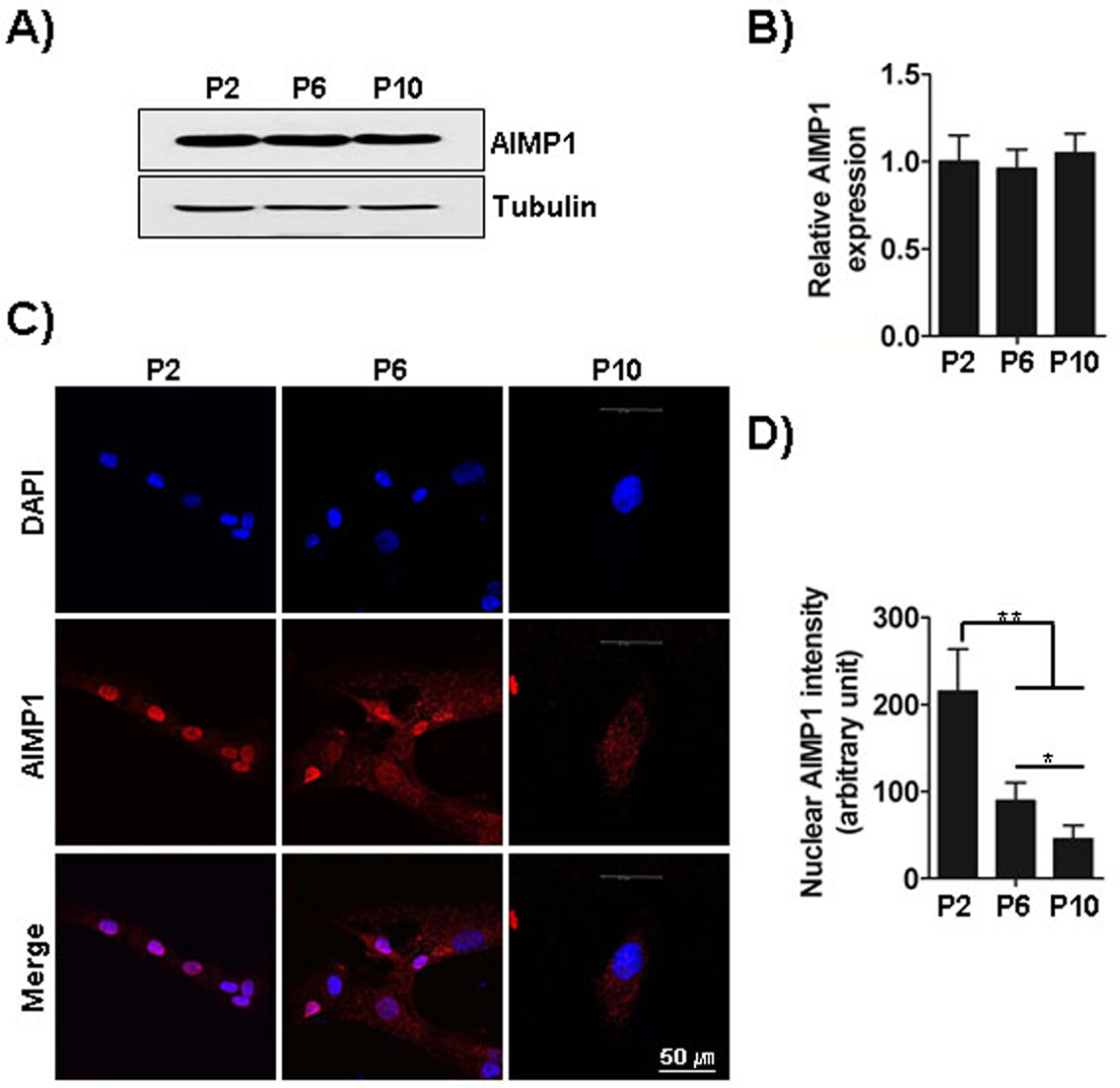

Supplement: Supplementary Figure 2 [file cddis201617x3.tif]

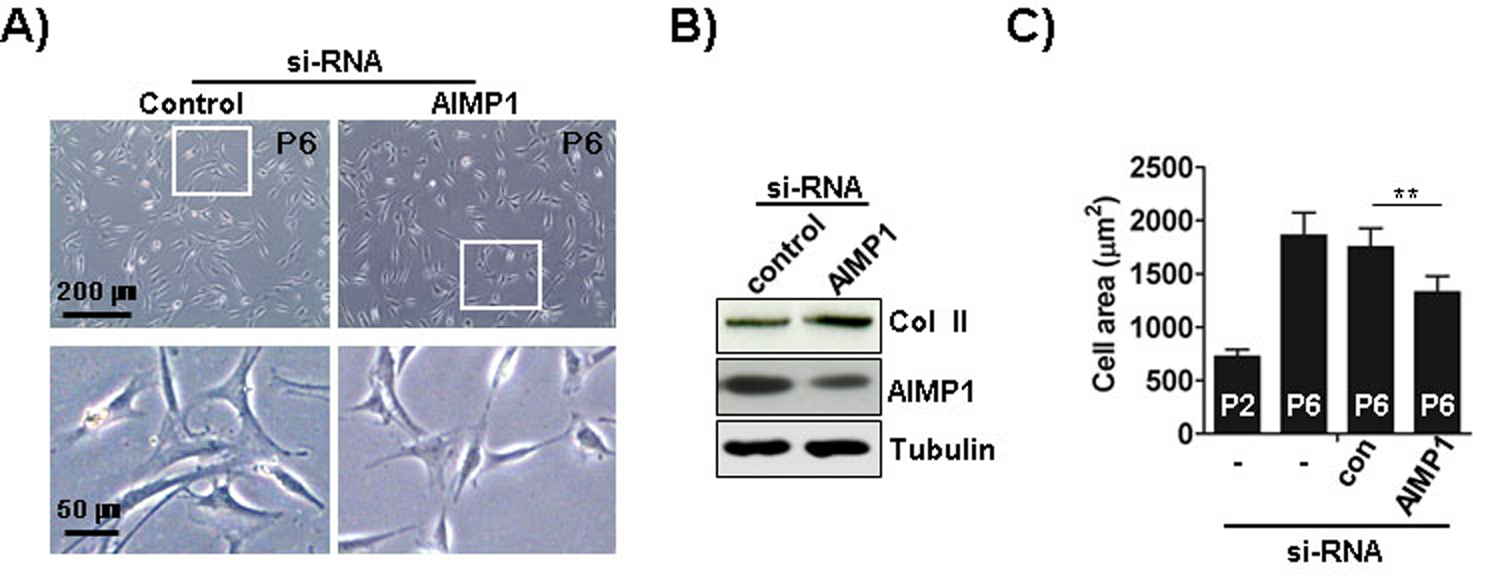

Supplement: Supplementary Figure 3 [file cddis201617x4.tif]

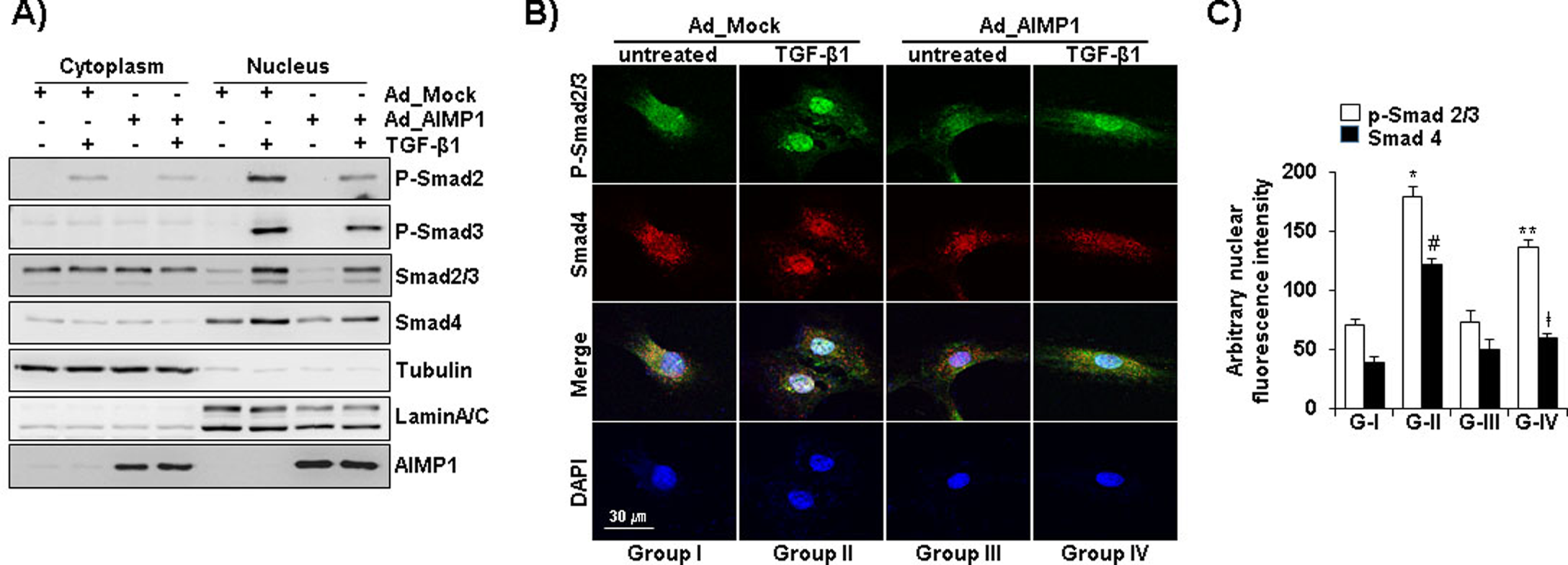

Supplement: Supplementary Figure 4 [file cddis201617x5.tif]

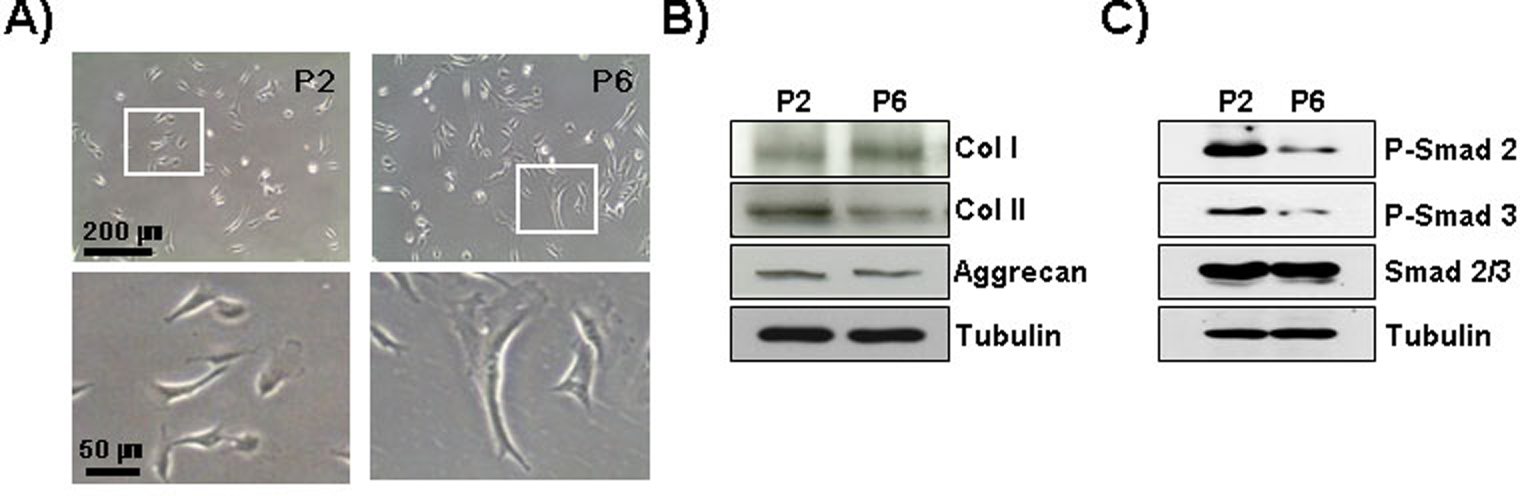

Supplement: Supplementary Figure 5 [file cddis201617x6.tif]
